# Effect of Chinese young children’s epidemic cognition on their coping behavior: mediating role of emotion

**DOI:** 10.1186/s40359-023-01106-5

**Published:** 2023-03-09

**Authors:** Yonggang Wei, Yu Shi, Qiao Zhou, Ruihan Zhu, Lu Wang

**Affiliations:** 1 Chongqing Early Childhood Education Quality Monitoring and Evaluation Research Center, Chongqing Normal University, No. 37 Middle Road, University Town, Gaoxin District, 401331 Chongqing, China; 2 The First Kindergarten of Chengdu, 610058 Sichuan, China; 3 Chengdu Qingyang Tianfu Kindergarten, 610014 Sichuan, China; 4 Chongqing Yubei Kindergarten, 401120 Chongqing, China

**Keywords:** COVID-19 pandemic, Young children, Epidemic cognition, Epidemic coping, Emotion

## Abstract

**Introduction:**

Young children’s epidemic awareness and risk prevention about public health emergencies such as the COVID-19 are issues of great importance deserving research.

**Objective:**

To explore the effect of young children’s epidemic cognition on their coping behavior, and the mediating role of emotion.

**Method:**

An online anonymous survey was administrated on 2221 Chinese parents of young children aged three to six during the COVID-19 overwhelming period.

**Results:**

(1) The epidemic cognition (*M* = 4.17, *SD* = 0.73), the coping behavior (*M* = 4.16, *SD* = 0.65), and the emotion (*M* = 3.99, *SD* = 0.81) were at a relatively high level. (2) Young children’s epidemic cognition significantly predicted their coping behavior (*β* = 0.71, *t* = 45.29, *P* < 0.001). The positive prediction effect of epidemic cognition on young children’s emotion was significant (*β* = 0.19, *t* = 8.56, *P* < 0.001), and emotion had a significant positive predictive effect on young children’s coping behavior (*β* = 0.20, *t* = 4.89, *P* < 0.001).

**Conclusion:**

Young children’s epidemic cognition can significantly predict their coping behavior, and emotion plays a significant mediating role in their relation. It is necessary for practitioners to optimize the contents and methods of epidemic education on young children.

## Introduction

### Young children’s vulnerability under public emergencies

Due to the unpredictability, high risk and extensive influence of the COVID-19 pandemic, the public has different degrees of anxiety, panic and other negative emotions [1, 2]. Young children’s coping behavior during the COVID-19 epidemic is essentially a decision-making behavior in a dangerous situation. They exhibit risk-seeking tendencies (e.g., playing in close quarters) or risk-averse tendencies (e.g., playing at home as much as possible) by making predictive judgments about risk factors in an event. Once they make wrong decisions or behave improperly, irreversible harm or even tragic consequences will be caused to them. Some studies pointed out that in most countries, accidental injuries posed a great threat to children’s health, and the wrong response behavior was the main cause of children’s death in accidents [3–5]. When young children are unsupervised, they are more likely to take risks to join in dangerous situations, and the chance of injury will also increase significantly [3, 6]. As a special group, young children are more vulnerable to major public emergencies such as the COVID-19 epidemic due to their lack of social experience and reasoning ability to deal with emergencies, as well as the ability to identify risks and protect themselves [7]. Therefore, it is very important to guide young children to correctly deal with the epidemic crisis and efficiently prevent them from the epidemic risk under the COVID-19 pandemic.

### Relations among epidemic cognition, coping behavior, and emotion experience

Epidemic risk cognition refers to individuals’ cognition and judgment of the possibility and harmfulness of epidemic risk events in uncertain situations. It is affected by the objective danger degree of the event, but it does not completely depend on the objective characteristics of the event [8]. People’s risk perception of crisis events involves two dimensions, familiarity and controllability, and “unknown and uncontrollable” events are often perceived as high-risk events [9]. People show more worries about the epidemic situation and perceive higher threats, and the higher the cognitive level of risks are [10, 11]. Coping style is the cognitive and behavioral style adopted by individuals when facing setbacks and pressures, also known as coping strategies or coping mechanisms, and it is an important factor in the process of psychological stress [12]. Children’s cognitive level will affect their prediction of risk probability, thus affecting their coping style [13]. Cognitive factors such as risk assessment, belief in the possibility of personal injury and attribution of injury (self, others and bad luck) will affect children’s coping style in risk situations [5].

Emotional experience is an important part of emotion, which refers to how individuals experience their emotions[14]. When individuals can experience diverse and well-differentiated emotions, they are considered to be able to show more empathy for others; at the same time, the more complex an individual’s emotional experience is, the more he can understand others’ feelings and have higher empathy [15]. Major public health emergencies can lead to certain acute psychological and social stress, and affect people’s physical and mental health and emotional experience [16]. Children tend to seek adventure when they make happy and excited expectations of dangerous activities; on the contrary, when children have negative expectations of fear and worry about dangerous activities, they will avoid such risky behaviors. This proves that expected emotion is the key predictor of children’s coping style [17]. In addition to expected emotions, immediate emotions can also affect coping styles. The Risk-as-Emotion model points out that both expected emotions and unconscious immediate emotions, which are influenced by cognitive assessment, will affect individual coping styles in different ways [18]. For example, in ultimatum task, children’s coping style will be influenced by emotional experience [19]. When children are satisfied with the distribution and have positive emotions in the game, they choose to accept the distribution. However, if they are dissatisfied with the distribution mode and have negative emotions, they will exercise veto power in the task.

Brain imaging research shows that cognition and emotion interact and connect with each other through the nerve center, thus contributing to a series of behaviors [20]. The two-process model also believes that children’s coping styles will be influenced by the interaction of receptive analytical systems and inspiring systems with stronger emotions [21]. According to the theory of emotional evaluation, when faced with a certain stress, an individual will automatically make a cognitive evaluation of the threats in environmental information, resulting in corresponding emotional responses and further triggering corresponding behaviors [22]. These studies show that strengthening individual’s cognitive level and keeping positive emotions are important strategies to ensure individual’s rational coping behavior [23, 24].

### Current study

In order to help young children to safely spend the hard time of the COVID-19 epidemic, it is necessary to investigate how they perceive the epidemic, what they experience, and how they cope with the crisis. However, in major emergent public health events such as COVID-19, few researchers have explored how young children’s cognition on the event and corresponding emotional experience affect their coping behaviors in existing literature. To explore the coping behaviors of young children and their influencing factors during the COVID-19 outbreak can provide a positive reference for coping with possible future public health emergencies. Therefore, on the basis of existing literature on young children’s risk perception [25], emotional experience [22], and the their relations to coping behavior [7, 19], this study is to explore the effect of young children’s epidemic cognition on their coping behavior and the mediating role of emotion. We propose the following assumptions: (1) because the society has carried out a lot of publicity and education during the COVID-19 outbreak period, young children’s perception of the pandemic and coping behavior may be satisfactory; (2) young children’s pandemic cognition can significantly predict their epidemic coping behavior; (3) emotion may play a significant mediating role in the relation between young children’s epidemic cognition and coping behavior.

## Method

### Participants

From February 3 to February 12, 2020 during the COVID-19 pandemic, an online anonymous questionnaire survey was conducted through the WenJuanXing public online platform in China. The entry criteria were as the following: (1) the COVID-19 pandemic was overwhelming at where they live; (2) the age of young children was from three to six; (3) parents filling out the questionnaire was staying together with their children, being familiar with their living state during the epidemic period. Except for the above entry criteria, the criteria for deleting invalid questionnaires were also included: (1) the questionnaires filled in by the subjects had obvious regularity; (2) the answers filled in by the subjects had multiple missing values; (3) the answers given by the same IP address were repeated. Random sampling was conducted for all provinces and cities in China, and finally 2,221 valid questionnaires were randomly collected from parents in 26 provincial areas of China. Among these young children, 1,136 (51.15%) were boys, and 1,085 (48.85%) were girls. There were 222 3-year-olds (9.99%), 399 4-year-olds (17.97%), 680 5-year-olds (30.62%), and 920 6-year-olds (41.42%).

### Measure

This questionnaire was compiled by referring to the related research of primary and secondary school students and preschool children’s disease cognition and prevention, combined with the performance of risk prevention and control of children in epidemic period. There were 28 questions in total, all of which were reported by parents of children. Young children’s parents and teachers were consulted to read through and modified the questions to make them suitable for young children’s daily life during the COVID-19 pandemic. In this questionnaire, the parents were asked to evaluate the children’s performance during the epidemic period. A five-point Likert scale was adopted, with 1–5 representing the range from “completely inconsistent” to “completely consistent”. The higher the score, the better young children’s cognition and coping behavior on the COVID-19 pandemic, and the more positive their emotional experience were.

Exploratory factor analysis was conducted, and the items were screened according to statistical standards. Finally, five factors (63.75% variation) were obtained, including COVID-19 knowledge, home protection knowledge, health care behavior, home-stay behavior, and positive and pleasant experience. We classified the COVID-19 knowledge factor and the home protection knowledge factor as the epidemic cognition subscale, the health care behavior factor and the home-staying behavior factor as the coping behavior subscales, and the factor of positive and pleasant experience as the emotion subscales.

The reliability coefficient of Cronbach’s alpha of the total questionnaire was 0.93, and the reliability coefficient of Cronbach’s alpha of each dimension was between 0.74 and 0.91, indicating that the questionnaire had good stability and internal consistency. The confirmatory factor analysis and fitting indicators were as the following:χ^2^/df = 9.199, GFI = 0.899, CFI = 0.923, IFI = 0.923, TLI = 0.913, RMSEA = 0.061. It indicated a good structural validity. According to the statistical results (see Table 1), there were significant positive correlations among the three dimensions with the coefficients between 0.19 and 0.70, and also significant positive correlations between all dimensions and the total questionnaire with the coefficients between 0.66 and 0.81, indicating that the direction of the questionnaire was consistent between all dimensions and the total questionnaire, with reliable structural validity.

**Table 1:** The mean, standard deviation and correlation of each variable

	*M*	*SD*	Epidemic cognition	Emotion	Coping behavior
Epidemic cognition	4.17	0.73			
Emotion	3.99	0.81	0.19^***^		
Coping behavior	4.16	0.65	0.70^***^	0.20^***^	
The whole questionnaire	4.11	0.55	0.81^**^	0.66^**^	0.81^**^

**P<0.01, ***P<0.001.

### Common method deviation avoidance

Anonymous measurement and partial item reversal were adopted to control the common method deviation. Harman single factor test and factor analysis were also used to test the common method deviation of the collected data. The KMO of the questionnaire items was 0.95 and the Bartlett value was 35921.24 (*P* < 0.001), which was suitable for factor analysis. Un-rotated exploratory factor analysis results extracted a total of 5 factors with characteristic roots greater than 1, and the maximum factor variance explanation rate was 39.39% (less than 40%), indicating that there was no serious common method bias in this study.

## Results

### Young children’s epidemic cognition, coping behavior, and emotion

As can be seen from Table 1, for the subscales of the questionnaire, the average values of the epidemic cognition (*M* = 4.17, *SD* = 0.73), the coping behavior (*M* = 4.16, *SD* = 0.65), and the emotion (*M* = 3.99, *SD* = 0.81) were all relatively high (full score of the mean is 5), indicating that the overall performance of Chinese young children during the epidemic was satisfactory. Chinese young children could better understand the basic epidemic knowledge, behaved well in risk prevention, and maintained positive emotions. This result is consistent with the hypothesis of this study.

In order to verify whether age and one-child status were influencing factors for Chinese young children’s epidemic awareness, ANOVA and t-test were used to compare the performance of young children. The ANOVA results showed that the performance of young children in epidemic period was better and better with increasing age (*F* = 54.90, *P* < 0.001). The t-test results showed that there was a significant difference (*t* = 4.42, *P* < 0.001) between the non-single-child group (*M* = 4.18, *SD* = 0.58) and the single-child group (*M* = 4.05, *SD* = 0.61). These differences suggest that the two variables (age and one-child status) are influencing factors for the performance of Chinese young children during the epidemic.

### Mediating role of emotion in the relation between young children’s epidemic cognition and coping behavior

Bootstrap method in Hayes’ PROCESS for SPSS (Bootstrap = 5000) was used to test the mediation model of emotion, and the results are shown in Tables 2 and 3.

**Table 2:** The mediation model of emotion

	Coping behavior					Coping behavior					Emotion						
	*B*	*t*	*P*		*SE*	*B*	*t*	*P*		*SE*		*B*	*t*	*P*		*SE*	
Age	-0.02	-1.67	0.10		0.01	-0.02	-1.54	0.12		0.01		0.02	1.12	0.26		0.02	
Single-child status	0.02	1.05	0.29		0.02	0.02	0.81	0.42		0.02		-0.09	-2.27	0.02		0.04	
Epidemic cognition	0.62	43.91	0.00		0.01	0.63	45.29	0.00		0.01		0.21	8.56	0.00		0.02	
Emotion	0.06	4.89	0.00		0.01												
*R* ^*2*^	0.50					0.50					0.04						
*F*	445.56^***^					545.34^***^					21.45^***^						

***P<0.001.

**Table 3:** Total effect, direct effect, and intermediate effect

	Effect size	Boot S.E.	Boot CI Lower limit	Boot CI Upper limit	Relative effect size
Total effect	0.63	0.02	0.72	0.80	
Direct effect	0.62	0.02	0.58	0.66	98%
Intermediate effect of emotion	0.01	0.004	0.01	0.02	2%

After controlling for age and one-child status, epidemic cognition significantly predicted young children’s coping behavior (*β* = 0.71, *t* = 45.29, *P* < 0.001), and the direct predictive effect of epidemic cognition on young children’s coping behavior was still significant when the mediating variable was included (*β* = 0.69, *t* = 43.91, *P* < 0.001). This shows that young children have been able to integrate knowledge and action during the epidemic. The positive prediction effect of epidemic cognition on young children’s emotion was significant (*β* = 0.19, *t* = 8.56, *P* < 0.001), emotion also had a significant positive predictive effect on young children’s coping behavior (*β* = 0.20, *t* = 4.89, *P* < 0.001).

In addition, the upper and lower bounds of the bootstrap 95% confidence interval of the direct effect of the epidemic cognition on young children’s coping behavior and the mediating effect of emotion did not contain the value of zero. This suggests that the epidemic cognition can not only directly predict young children’s coping behavior, but also indirectly predict young children’s coping behavior through the mediating effect of emotion. Furthermore, the epidemic cognition had a stronger impact on young children’s coping behavior than that on the emotion. These results have verified our hypothesis.

## Discussion

### Young children’s risk prevention during the COVID-19 pandemic

The results show that young children perform well in the epidemic cognition, emotional experience, and coping behavior, and have a good sense of risk prevention. While home isolation may be easy for adults to adjust, it is not easy for young children to cope with. Why do young children suppress their instincts and peacefully stay home? An earlier study found that young children could already judge risk factors in a situation [26]. When they estimate that the danger of the situation is too high and will easily lead to their own injury, they tend to choose risk avoidance [27]. In addition, we speculate that parental and other relatives guidance will also be an important factor in raising cognition of risk in young children during the COVID-19 outbreak. According to the domain-specific theory of risk decision making, individual risk preference is not stable and will be affected by domain-specific situations, thus showing different risk tendencies [28]. In the special situation of the whole population staying at home during the COVID-19 pandemic, parents undoubtedly become an important factor affecting the risk tendency of young children. A large amount of evidence has shown that parents, as close contacts of children, have a great influence on children’s cognition, behavior and emotion [29, 30]. If parents are good at teaching safety knowledge and rules to children, children will be more inclined to avoid risks and conduct safe behaviors [31]. However, for young children, they are unable to fully and accurately predict the outcome of behavior and choose activities that can exercise themselves without injuries, which sometimes leads to poor decision-making by the children in independent activities [4, 32]. This reflects that the effects of risk prevention education on children vary with their understanding and compliance. Even so, for young children with poor reasoning and judgment ability, risk prevention education is still the most effective way to promote rational decision-making.

### Social referential effect in young children’s epidemic coping

It has been found that the performance of non-single-child group in terms of epidemic cognition, emotional experience and coping behavior are significantly better than that of the single-child group. In fact, in other research areas, researchers have also found that sibling attitudes or behaviors significantly predict children’s decision-making behavior, including eating behavior [33], consumption behavior [34], watching TV [35], and other aspects of our lives [27].This may be because the children in non-single-child families tend to make social comparisons with their brothers and sisters, and the status of these intimate others can easily affect their decision-making behavior, thus producing social referential effect. According to the social reference point theory, in order to make more accurate self-assessment, individuals prefer to judge their positions relative to others [36, 37]. Like others, one himself or herself is social neutral; when they are better than others, they will achieve social gain; and social loss is when one is not as good as others [38]. Social loss situations usually stimulate individuals’ negative emotions such as inferiority and loss, which will stimulate a series of behaviors, such as rejecting those who perform better or seeking ways to perform better, or even willing to take risks in order to get rid of social loss [37]. On the contrary, when the society benefits, they always have a positive self-evaluation of themselves, leading to positive emotional experience, so that people can better deal with some negative events [36]. Therefore, if sibling children have a stronger sense of risk prevention and show social benefits in risk decision-making, they will perform better in risk prevention activities, so as to maintain the stability of self-concept and ensure that they are in a good psychological state.

### Emotion mediating in epidemic cognition and coping behavior

This study has found that epidemic cognition has a significant positive predictive effect on young children’s coping behavior. This is consistent with the results of adult subjects during the epidemic [39]. Through further research, it is found that epidemic cognition can also indirectly affect children’s coping behaviors through the mediation of emotions. There may be two explanations for this result. First, epidemic cognition can positively predict individual emotions. After a survey of the German public in March 2020, Jungmann et al. found that individuals’ knowledge of COVID-19 had an impact on their emotions; specifically, the more they knew about the epidemic, the more optimistic their emotions were [40]. The risk communication theory can explain this phenomenon, which points out that the more sufficient the relevant information which the public can obtain in a risk event, the more beneficial it is for them to make an objective interpretation of the event nature, thus reducing their psychological burden [41]. Secondly, emotions influenced by the COVID-19 perception will further influence coping behavior. From the prospect theory, to the regret theory and subjective anticipatory pleasure theory, they all expounded the influence of cognition and emotion on decision making [18, 42]. Regarding children’s risk-taking behaviors in the context of games, Morrongiello and Matheis found that when children were provided with enough risk information to help them recognize the risks in the environment, they were more likely to worry and fear about the behavioral results after taking risks, and then tended to avoid risks [17].

In addition, this study has found that the direct effect value of epidemic cognition on coping behavior is much higher than the indirect effect value while emotion mediating. In other words, epidemic cognition is a better predictor for young children’s coping behavior during public health emergencies than emotion. This is consistent with the results of previous studies [13]. For example, Li et al. found that cognitive system and emotion-affective system could jointly affect children’s behavioral decision-making, but the relation between cognitive system and decision performance was closer than that between emotional systems [43]. Webb et al. found that in the Iowa gambling task, the individual’s cognitive system could better explain the differences in individual decision performance than the emotional and feeling system [44].

## Implications for practice

This study has found that the direct effect value of COVID-19 cognition on children’s coping behavior was very high, indicating that the knowledge of COVID-19 epidemic is helpful to improve children’s self-protection ability and actively avoid risks. In addition, the results also showed that children generally had a high level of cognition of COVID-19 during the epidemic, and they had been able to understand the basic characteristics of the epidemic, the degree of danger, prevention methods and other common knowledge. According to the theory of proximal development zone, educators should focus on children’s current development level, adjust the content and way of education, stimulate children’s interest in learning to make it more effective. For this reason, the education about epidemic after the resumption of schools should not only focus on simple knowledge of epidemic prevention and health, but should respond to the impact of the epidemic and make full use of educational materials in the process of fighting the epidemic. For example, kindergartens can carry out educational activities on the theme of “What is COVID-19?“, “Don’t come here with the virus”, “Charm of Nature”, “Loveliest person” and “My participation in the fight against the epidemic” to cultivate children’s awareness of life, patriotism, environmental protection and responsibility while mastering the knowledge and skills of epidemic prevention. Secondly, educational methods should not only be limited to simple oral preaching, but should be combined with children’s own experience and interest needs to make educational activities game-like, life-like and practical. For example, in role games, educators can encourage children to play the role of the person they most admire during the pandemic and cultivate their sense of responsibility. In the daily routine education, children can create children’s songs together or create the environment together with children to remind and internalize children to develop good living and health habits, so that physical exercise, reasonable diet and disease prevention become normal.

This study has found that in addition to COVID-19 cognition, children’s emotional experience in crisis also affected their coping behaviors. However, the current epidemic prevention education for children generally ignores the training of children’s emotional regulation ability. Therefore, in sudden public health events, we should carefully observe and record children’s emotional fluctuations in the event, through children’s expressions, actions, language and other explicit behaviors to visit children’s psychological changes, timely communicate with children the cause of negative emotions, so that children learn to express emotions. In addition, we should also guide children through a reasonable way to vent bad emotions. For example, as the temperature rises, wearing a mask will feel more and more uncomfortable, and it is easier for children to become sensitive and irritable. In this case, educators can take children to outdoors, remove masks and have some sports. In addition, hero education under the epidemic is important, but it is also necessary to prevent children from being too immersed in empathy and causing excessive anxiety. To this end, educators can fully tap network resources, through video, picture books, electronic picture books and other ways to convey positive energy information to children, enhance children’s confidence to overcome difficulties and expectations for a better life.

When the sample data were collected, this study was in the critical period of home epidemic prevention, so the research perspective mainly focused on parents’ epidemic prevention education for children, but the environment outside the family was full of potential crises. As a susceptible population to epidemics, young children are more vulnerable to the threat of epidemics in crowded and closed places. Kindergartens are special places with a high concentration of susceptible populations, and common infectious diseases such as scarlet fever, hand, foot and mouth disease, and chicken pox have a high prevalence rate in kindergartens. Therefore, kindergartens should pay attention to the prevention of infectious diseases education for children, and through home cooperation to jointly cultivate children’s awareness of infectious diseases prevention and self-protection ability. For example, parents can be guided to actively participate in infectious disease education activities in kindergartens, which is not only conducive to expanding the knowledge reserve of parents on epidemic prevention, but also enables parents to enhance the ability of early childhood education in practice. At the same time, we should fully tap community education resources and expand education space. For example, in the community, children work as the main body, and parents help them carry out parent-child infectious disease outdoor publicity activities, encourage children to spread infectious disease knowledge to community residents. In short, we should fully integrate and play the positive role of kindergarten, family and community to provide necessary environmental support, material support and psychological support for children.

## Conclusion

Chinese young children’s cognition and coping behavior of COVID-19 epidemic are at a satisfactory level. Chinese young children’s cognition of COVID-19 epidemic can significantly predict their coping behavior. Emotion plays a significant mediating role in the relationship between Chinese young children’s cognition and coping behavior of COVID-19 epidemic.

## Data Availability

The datasets used and/or analyzed during the current study available from the corresponding author on reasonable request.
